# CD4+ T-lymphocyte telomere length is related to fibrosis stage, clinical outcome and treatment response in chronic hepatitis C virus infection

**DOI:** 10.1016/j.jhep.2010.03.005

**Published:** 2010-08

**Authors:** Matthew Hoare, William T.H. Gelson, Abhi Das, Jean M. Fletcher, Susan E. Davies, Martin D. Curran, Sarah L. Vowler, Mala K. Maini, Arne N. Akbar, Graeme J.M. Alexander

**Affiliations:** 1 Department of Medicine, School of Clinical Medicine, University of Cambridge, Cambridge, UK; 2 Department of Immunology, University College London, London, UK; 3 Department of Pathology, University of Cambridge, Cambridge, UK; 4 Clinical Microbiology and Public Health Laboratory, Health Protection Agency, Addenbrooke’s Hospital, Cambridge, UK; 5 Centre for Applied Medical Statistics, Department of Public Health and Primary Care, University of Cambridge, Institute of Public Health, Forvie Site, Robinson Way, Cambridge, UK

**Keywords:** HCV, hepatitis C virus, HCC, hepatocellular carcinoma, CMV, cytomegalovirus, HBV, hepatitis B virus, EBV, Epstein–Barr virus, HIV, human immunodeficiency virus, IFN-α, interferon-α, PBMCs, peripheral blood mononuclear cells, APCs, antigen presenting cells, HR, hazard ratio, Hepatitis C, Telomere, T-lymphocyte, Immune senescence, Human, Ageing, Hepatocellular carcinoma, Outcome study, Interferon-α

## Abstract

**Background & Aims:**

Increasing age is associated with impaired immune function and in chronic HCV infection specifically, with progressive fibrosis, liver failure, HCC and impaired responses to antiviral therapy. T-lymphocyte telomere length declines with age. We hypothesised that shorter T-lymphocyte telomere length would be associated with poor clinical outcome in HCV infection.

**Methods:**

Circulating T-lymphocyte telomere length, an objective measure of immune senescence, was measured by flow-FISH in 135 HCV-RNA-positive, treatment-naïve patients and 41 healthy controls in relation to clinical outcome.

**Results:**

Shorter CD4+CD45RO+ T-lymphocyte telomeres were associated with severe fibrosis (*p *= 0.003), independent of male sex (*p *= 0.04), CMV positivity (*p *= 0.003), previous HBV infection (*p *= 0.007), and age (*p *= ns) in viraemic patients compared to controls. There were inverse correlations between CD4+CD45RO+ telomere length and fibrosis stage (*p *<0.001), portal tract inflammatory grade (*p *= 0.035), prothrombin time (*p *<0.001) and bilirubin (*p *= 0.001). One hundred and twenty-four viraemic individuals were followed prospectively to a composite endpoint of death, hepatic decompensation or HCC. Independent of age, those with shorter CD4+CD45RO+ telomeres were less likely to be complication free after 2-years than those with longer telomeres (86% versus 96%, *p *= 0.009) with an age-adjusted hazard ratio of 0.93 (0.90–0.96). In addition, CD4+CD45RO+ telomere length predicted successful antiviral therapy (*p *= 0.001) independent of other factors.

**Conclusions:**

CD4+ T-lymphocyte telomere length, independent of age, was related to inflammatory grade, fibrosis stage, laboratory indices of severity, subsequent hepatic decompensation and treatment outcome in patients with chronic HCV infection.

## Introduction

Chronic hepatitis C virus (HCV) infection may lead to cirrhosis and hepatocellular carcinoma (HCC). Prospective cohort studies demonstrate that the majority of viraemic individuals never develop severe hepatic fibrosis [1,2]; factors associated with progressive fibrosis include male sex, obesity and concurrent alcohol misuse [3]. Several groups have demonstrated that long-term prognosis in HCV infection is critically dependent upon age at acquisition [3–6].

Human ageing is associated with impaired adaptive immunity [7,8] with reduced numbers of naïve cells, increased numbers of antigen-experienced cells and oligoclonal expansion of CD8+ T-lymphocytes. Prospective studies demonstrate that these changes predict morbidity and mortality in healthy elderly populations, leading to the concept of an ‘immune risk’ phenotype [8–10].

Progressive shortening of lymphocyte telomeres is characteristic of immune senescence and may underpin changes in lymphocyte function [11]. Telomeres are repeating sequences of DNA at the extreme ends of eukaryotic chromosomes. DNA polymerase cannot transcribe to the chromosomal tip; thus telomeres shorten with each cell division, leading eventually to a DNA damage signal mediated by γ-H2AX, p53 and p16^INK4A^ [12,13] preventing further mitoses [14], a point defined as replicative senescence. In healthy elderly subjects, shortened lymphocytic telomeres were associated with increased mortality, particularly after infection [9].

Chronic viral infections accelerate immune senescence and declining immune function [15,16]. Cytomegalovirus (CMV) infection is implicated in accelerated immune senescence within both CMV-specific and non-specific lymphocytes [17] and is associated with oligoclonal expansion of CD4+ and CD8+ lymphocytes in elderly populations [18]. Oligoclonality of CD8+ lymphocytes in HCV infection is associated with increased fibrosis and reduced responses to antiviral therapy [19]. Kitay-Cohen et al. demonstrated that viraemic HCV is associated with short peripheral leucocyte telomeres, although no relation to clinical or treatment outcome was explored [20].

The marked effect of age on the natural history of HCV infection and the response to antiviral therapy is unexplained [21,22]. This study addresses the relation between lymphocyte telomere length, age and clinical outcome in chronic HCV infection.

## Materials and methods

HCV infected patients recruited at Addenbrooke’s Hospital, Cambridge gave written informed consent with approval of the Local Research Ethics Committee. Patients co-infected with HIV, HBV or other chronic liver disease, identified by history, blood tests or liver biopsy, were excluded. Lymphocytes from healthy controls were obtained from local volunteers; none gave a history of chronic illness or intravenous drug usage. Study subject groups were defined as: healthy controls; viraemic with mild disease (Ishak fibrosis 0–3); viraemic with severe disease (Ishak fibrosis 4–6) (Table 1).

IgG antibody against CMV was sought by chemiluminescent immunoassay (Diasorin, Saluggia, Italy). Anti-HCV IgG was sought by ADVIA Centaur sandwich immunoassay (Bayer, Tarrytown, NY); HCV-RNA by real-time taqman PCR assay targeting the conserved 5′ non-coding region on a Rotor-gene™ 3000 instrument (Corbett Lifescience, Sydney, Australia). Probit analysis (Stats Direct, www.statsdirect.com) revealed a lower limit of detection of 25 IU/ml (6.3–38.6, 95% confidence intervals). Patients with anti-HCV were defined as viraemic if HCV-RNA was detected on all 3 occasions at 6-month intervals; only those with a liver biopsy within 2 years of study entry were included. Those with inconsistent HCV-RNA results were excluded. All subjects were treatment-naïve at enrolment and none had evidence of current or previous hepatic decompensation or HCC. Less than 20% of HCV-infected subjects had a known, single time of infection; thus duration of infection was unavailable.

Liver biopsies were scored according to Ishak criteria by a specialist liver histopathologist (SED), blinded to the lymphocyte studies or outcome. Histologic activity index represented the sum of interface hepatitis (scored 0–4), confluent necrosis (0–6), lobular inflammation (0–4) and portal tract inflammation (0–4). Fibrosis was staged 0 (absent) to 6 (cirrhosis). Steatosis was scored 0–3.

A subgroup of patients was treated subsequently with peg-IFN-α2a and ribavirin (RBV) (Roche, Welwyn Garden City, UK). All patients received 180 mcg of peg-IFN-α2a sub-cutaneously once weekly, with subsequent dose-alterations dictated by clinical and laboratory parameters and RBV (800 mg daily for genotype 2 or 3 or weight-based between 1000 and 1200 mg for genotype 1 or 4). Patients with genotype 1 or 4 were treated for 12 weeks and then a further 36 weeks if they were HCV-RNA negative or had undergone a 2 log_10_ drop in viral load compared to baseline. Those with genotype 2 or 3 infection were treated for 24 weeks. All subjects underwent HCV-RNA testing six months after cessation of therapy to determine if they had achieved sustained virological response (SVR). No patient received growth factors to support haematological parameters. Patients unable to tolerate antiviral therapy on symptomatic grounds were excluded from analysis.

A composite endpoint was used for outcome analysis: outcome events were death, first episode of hepatic decompensation (new rise in bilirubin to twice the upper limit of normal, development of ascites, hepatic encephalopathy, portal hypertensive haemorrhage) or development of hepatocellular carcinoma. Outcome was determined from study entry and survivors were censored at last clinic appointment.

### Lymphocyte preparation

Peripheral blood mononuclear cells (PBMCs) were obtained by centrifugation of citrated blood over Lymphoprep (Nycomed, Roskilde, Denmark) and analysed immediately or cryopreserved at −80 °C in 80% foetal calf serum (Biosera, East Sussex, UK), 10% RPMI-1640 (Gibco, Paisley, UK) and 10% dimethyl sulphoxide (DMSO) (Sigma–Aldrich, Gillingham, UK).

### Flow cytometry

Flow-cytometric analysis of T-lymphocytes was performed using combinations of the following: CD4-biotin (Beckman Coulter, Fullerton, CA), CD4-PE-Cy5, CD8-biotin, CD8-PE-Cy5, CD27-APC, CD57-biotin (all BD, San Diego, CA), CD45RO-biotin (Ebiosciences, San Diego, CA), CD45RO-FITC (Dako, Glostrup, Denmark). Biotinylated antibodies were followed by streptavidin-Cy3 (Cedarlane laboratories, Ontario, Canada). All cytometry was performed on a FACScalibur analyser (BD) unless otherwise stated; data were analysed with FCSpress software (www.fcspress.com).

### Telomere length by flow cytometry

Telomere length of CD4+ or CD8+ T-cells was measured using 3-colour flow-FISH assay as described [23,24]. CD4+ PBMCs from one healthy individual were analysed in every experiment as an internal control. Lymphocyte telomere length within an individual is expressed as mean fluorescence intensity (MFI). Inter-experimental standard deviation for the same sample was 1.2% and 0.9% for CD4+ and CD8+ lymphocytes, respectively.

### Statistics

Population data were subjected to non-parametric analysis, with lymphocyte surface phenotype analysed by Kruskal–Wallis. Ishak scores are non-linear variables and therefore, associative data were analysed by Spearman’s Rank correlation coefficient. Univariate analysis of survival was performed by the Kaplan–Meier method; curves were compared with the log-rank method and hazard ratios constructed from a Cox regression analysis (Prism 5.0, Graphpad, San Diego, CA).

Backward stepwise multinomial regression analysis was performed to identify predictors of severe fibrosis using SPSS 15.0 for Windows, with allocation to the severe fibrosis group (Ishak fibrosis stage 4–6) as outcome. Input variables were gender, IgG anti-CMV status, age, BMI, IgG anti-HBc status, CD4+CD45RO+ telomere length and CD8+CD45RO+ telomere length.

To study factors associated with SVR, logistic regression analysis was performed using SPSS 15.0 for Windows, with SVR as outcome. Input variables were age, sex, CMV antibody status, HCV genotype, Ishak fibrosis score, body mass index (BMI), CD8+CD45RO+ and CD4+CD45RO+ telomere length.

Only variables with a *p* value of <0.10 on univariate analysis were subjected to multi-variate analysis. *p* values of <0.05 were considered significant.

## Results

### T-lymphocyte telomere length, viraemia and fibrosis stage (Fig. 1)

Increasing age was associated with shortened CD4+ T-lymphocyte telomeres in healthy subjects (*p *= 0.015, Rs = −0.379) and HCV-infected groups (*p *<0.0001, Rs = −0.415). Study subjects were recruited according to HCV RNA status and hepatic fibrosis stage, so there were predictable differences between patient groups and controls with respect to CMV and IgG anti-HBc status (Table 1) and within patient groups for age. Both CD4+CD45RO+ and CD8+CD45RO+ T-cell telomere length were significantly shorter in HCV-infected subjects with severe fibrosis compared to controls and subjects with mild fibrosis.

To study the independent association between lymphocyte telomere length and fibrosis group a backward stepwise multinomial regression model was constructed to determine which factors were predictive of severe fibrosis. Input variables into the model were gender, IgG anti-CMV status, age, BMI, IgG anti-HBc status, CD4+CD45RO+ telomere length and CD8+CD45RO+ telomere length.

Backward stepwise multinomial regression demonstrated that male sex (OR; 95% CI; *p* value) (2.84 (1.07, 7.53); *p *= 0.04), CMV positivity (4.18 (1.63, 10.73); *p *= 0.003), anti-HBc positivity (4.74 (1.53, 14.72); *p *= 0.007) and decreasing CD4+CD45RO+ telomere length (1.05 (1.02, 1.78); *p *= 0.003) were independently associated with severe HCV-related fibrosis (Table 2).

Age and CD8+CD45RO+ telomere length showed significant association on simple regression analysis with severe fibrosis, but no independent association in backward stepwise regression analysis.

### Telomere length and cells with ‘advanced phenotype’

There were no differences in the proportion of CD4+ T-cells that were CD27+CD45RO− (*p *= 0.07), CD27+CD45RO+ (*p *= 0.14) or CD27−CD45RO+ (*p *= 0.44). Nor were there differences between the three groups in the proportion of CD8+ T-cells that were CD27+KLRG1−CD57− (Kruskal–Wallis, *p *= 0.21), KLRG1+CD57− (*p *= 0.48), or KLRG1+CD57+ (*p *= 0.58). Thus, shortened telomeres in chronic HCV infection were not attributable to accumulation of lymphocytes with ‘advanced phenotype’.

### Circulating lymphocyte telomere length, fibrosis, and portal inflammation (Fig. 2)

**Fig. 2:**
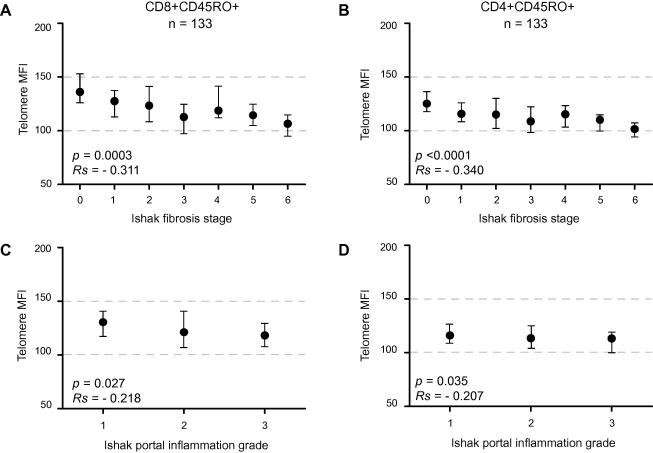
**Correlation of telomere length of (A and C) CD8+CD45RO+ or (B and D) CD4+CD45RO+ lymphocytes from 133 viraemic HCV subjects with (A and B) fibrosis stage or (C and D) portal tract inflammation grade**. Symbols and bars represent median and inter-quartile range; correlation by Spearman’s Rank.

The relation between CD4+ or CD8+ lymphocyte telomere length and histology was investigated in 133 viraemic patients. CD4+CD45RO+ lymphocyte telomere length correlated with fibrosis stage (*p *<0.001, Fig. 2B), portal tract inflammation grade (*p *= 0.035) and confluent necrosis (*p *= 0.036), but there was no evidence of correlation with interface hepatitis (*p *= 0.34), lobular hepatitis (*p *= 0.84) or steatosis (*p *= 0.76). CD8+CD45RO+ lymphocyte telomere length also correlated with fibrosis stage (*p *<0.001), portal tract inflammatory grade (*p *= 0.027) and confluent necrosis (*p *= 0.039), but showed no evidence of correlation with interface hepatitis (*p *= 0.125), lobular hepatitis (*p *= 0.504) or steatosis (*p *= 0.706).

### Lymphocyte telomere length and clinical parameters (Fig. 3)

The relation between CD4+ or CD8+ lymphocyte telomere length and clinical severity was investigated in 133 viraemic patients. There was a correlation between CD4+CD45RO+ telomere length and bilirubin (*p *<0.001), prothrombin time (*p *= 0.001) but not ALT (*p *= 0.08). There was also a correlation between CD8+CD45RO+ telomere length and bilirubin (*p *= 0.002), prothrombin time (*p = *0.004), but not with serum ALT (*p *= 0.12). These correlations remained significant after exclusion of outlying values.

### Short CD4+ lymphocyte telomere length and poor clinical outcome (Fig. 4 and Table 3)

Eleven patients were lost to follow-up. The remaining 124 viraemic subjects (without previous decompensation or elevated bilirubin) were followed prospectively for a median 724 days (IQR: 533–906). The cohort was divided into telomere length that was longer or shorter than the cohort median (111.9 for CD4+CD45RO+ and 118.8 for CD8+CD45RO+).

The proportion free of complication at 2 years was 96% and 86% for longer and shorter CD4+CD45RO+ telomeres, respectively (*p = *0.009); and 94% and 88% for longer and shorter CD8+CD45RO+ telomeres, respectively (*p *= 0.039). Utilising a proportional hazards model to investigate telomere length as a predictor of outcome, independent of age, demonstrated that a single point increase in CD4+CD45RO+ telomere MFI was associated with a HR of 0.93 (0.91–0.96) and 0.96 (95% CI: 0.93–0.99) in the CD8+CD45RO+ subset.

The findings were more marked when the analysis was restricted to viraemic subjects with severe fibrosis (*n* = 55, median follow-up 742 days (333–913 days) using cohort medians of 108.3 and 116.1, respectively); the proportion free of complications at 2 years was 97% and 70% for longer and shorter CD4+CD45RO+ telomeres, respectively (*p *<0.001) and 86% and 80% for longer and shorter CD8+CD45RO+ telomeres, respectively (*p *= 0.27). The age-adjusted HRs for CD4+CD45RO+ and CD8+CD45RO+ telomeres and the development of the composite outcome were 0.93 (0.90–0.96) (*p *= 0.02) and 0.96 (0.93–0.99) (*p *<0.001), respectively.

Analysis of each outcome independently in those with severe fibrosis (*n* = 55) revealed that longer CD4+ telomere length was associated with reduced evolution to HCC [*p *= 0.003, HR 0.92 (0.87–0.97)] and fewer episodes of hepatic decompensation [*p *= 0.003, HR 0.93 (0.89–0.98)]. No separate outcome was associated with shorter CD8+CD45RO+ telomere length: HCC (*p *= 0.1); decompensation (*p *= 0.05); death (*p *= 0.95) in those with severe fibrosis (data not shown).

**Fig. 4:**
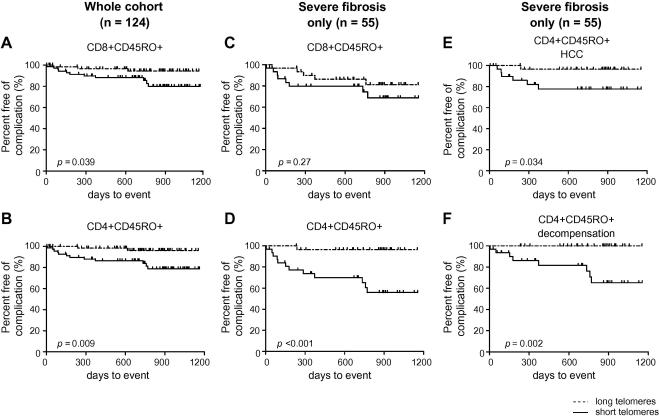
**Clinical outcome of viraemic subjects (*n* = 124) (A and B) or viraemic patients with severe fibrosis only (*n* = 55) (C and D) divided into subjects with telomeres longer (dashed line) or shorter (solid line) than the median. Kaplan–Meier analysis from study entry to outcome or censor date by log rank test: (A and C) CD8+CD45RO+ telomere length and (B and D) CD4+CD45RO+ telomere length. CD4+CD45RO+ telomere length from subjects with severe fibrosis (*n* = 55) analysed with each outcome independently: (E) Development of HCC; (F) Episode of decompensation**.

CD4+ telomere length as either a categorical (Fig. 4) or a continuous variable (Table 3) was related to the subsequent clinical course, independent of age and fibrosis stage.

### Baseline T-lymphocyte telomere length was related to SVR (Fig. 5 and Tables 4 and 5)

85 individuals (Table 4) treated with IFN-α and RBV were followed to 6 months after completing treatment; 38 subjects (45%) achieved SVR. Baseline CD4+CD45RO+ telomere length was longer in those achieving SVR compared to those who remained viraemic: 119.0 (107.8–126.8) and 105.5 (99.4–114.2), respectively (*p *<0.001).

**Supplementary Fig. 1:**
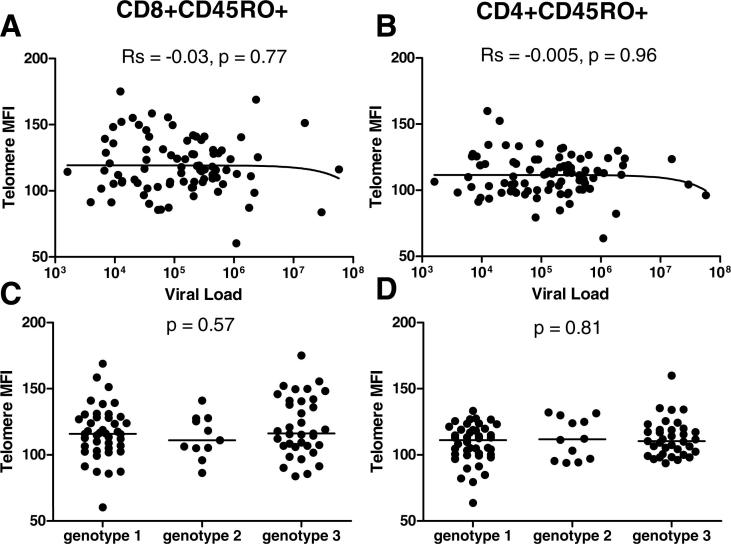
Baseline T-lymphocyte telomere length in (A and C) CD8+CD45RO+ and (B and D) CD4+CD45RO+ subsets in relation to HCV viral load (A and B) or viral genotype (C and D) (*n* = 85). Analysis by Spearman’s Rank correlation and Kruskal–Wallis test.

Similarly, CD8+CD45RO+ telomere length was significantly longer in those achieving SVR compared to those who did not: 128.1 (113.4–142.3) and 112.5 (104.5–118.2), respectively (Mann–Whitney, *p *< 0.001). There was no clear association between CD8+CD45RO+ or CD4+CD45RO+ telomere length and either viral load or viral genotype (Supplementary Fig. 1).

A multi-variate model was constructed to investigate whether baseline T-lymphocyte telomere length predicted SVR, independent of other factors known to predict treatment success. Input variables included age, sex, viral load, HCV genotype, Ishak fibrosis score, body mass index (BMI) and CD8+CD45RO+ or CD4CD45RO+ telomere length. The outcome measure was SVR.

Utilising backwards stepwise regression to remove non-significant variables viral load (*p *= 0.02) and CD4+CD45RO+ telomere length (*p *= 0.001) were associated independently with SVR. For each one point increase in CD4+CD45RO+ lymphocyte telomere MFI the odds ratio of SVR was 1.08 (1.03–1.13).

## Discussion

**Supplementary Fig. 2:**
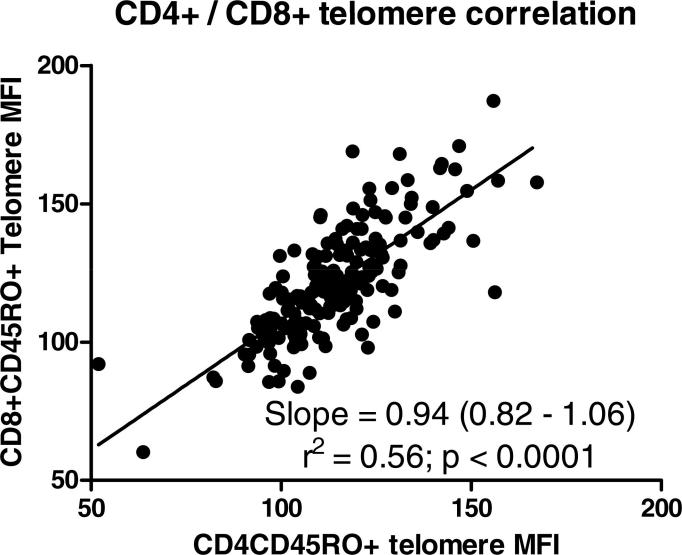
Correlation between CD4+CD45RO+ and CD8+CD45RO+ baseline T-lymphocyte telomere length in viraemic HCV patients (*n* = 133). Slope and 95% CI, goodness of fit by *r*^2^.

T-lymphocyte telomeres shortened with age in all groups, as anticipated [11]. T-cells from patients exposed to HCV had telomeres that were shorter than matched healthy controls with an overall difference in telomere length equivalent to 10-years additional ageing. The changes in T-cell telomere length were even more marked in those with severe fibrosis, equivalent to 15-years additional ageing. The findings associated with HCV infection were independent of factors known to influence immune senescence, including age, sex and CMV status. The changes were similar in both CD4+ and CD8+ T-lymphocytes, but the important association was between CD4+ telomere length and severe fibrosis. The apparent associations of CD8+ telomere length were due to their significant association with CD4+ telomere length (Supplementary Fig. 2). Importantly, by including age and telomere length in the three analyses of hepatic fibrosis progression, clinical outcome and treatment outcome, it is clear that telomere length is a significant predictor of all three, independent of patient age.

An important relation between immune senescence and clinical outcome was supported by the findings in cross sectional analysis that CD4+ T-lymphocyte telomere length correlated closely with increased fibrosis stage, increased grade of portal tract inflammation, prolonged prothrombin time and increased bilirubin, all factors that predict morbidity and mortality in patients with chronic HCV infection [3]. In a prospective study, despite the short duration of follow-up, patients with shorter CD4+ telomeres have outcomes inferior to those with longer telomeres, with more frequent progression to the composite endpoint of death, first episode of hepatic decompensation or hepatocellular carcinoma.

This study could not address whether HCV infection causes accelerated telomere shortening, whether individuals with shorter T-cell telomeres are pre-disposed to cirrhosis or whether lifestyles associated with HCV infection affect telomere length. Longitudinal follow-up is underway and may address these issues. Similarly, we have restricted the investigations of the relationship of lymphocyte telomere length and clinical outcome to HCV infection only. Previous studies have suggested that hepatocyte telomere shortening occurs in a variety of chronic liver diseases [25,26]. We hypothesise that lymphocytic telomere length shortening would occur in other chronic liver diseases, but no data yet exists to support this.

Our data pose the intriguing question of why telomere shortening occurs in T-cells in chronic HCV infection. There is no evidence of a failure to induce telomerase, an enzyme that maintains telomere length, in peripheral lymphocytes (unpublished data). The most likely explanation is chronic hepatic inflammation, with attendant cytokine release, leading to a bystander effect upon lymphocytes as observed in CMV infection [17] where both CMV-specific and non-specific cells develop accelerated senescence. One obvious issue is that of telomere length in HCV-specific cells. Multiple studies have demonstrated that such cells are detected rarely in the periphery [27,28] and thus cannot account for our findings. Their scarcity has also rendered it impossible to analyse telomere length in HCV-specific CD8+ or CD4+ populations with confidence.

Global lymphocyte telomere shortening has been observed previously and linked to poor clinical outcome in healthy elderly individuals [9], particularly that due to infection. Similarly, oligoclonality of the peripheral lymphocyte compartment has been associated with premature ageing of the immune system and increased morbidity [8]. Manfras et al. demonstrated that increased oligoclonality of the peripheral lymphocyte compartment was associated with poor responses to antiviral therapy in chronic HCV [19] and that individuals with increased CD8+ lymphocytes bearing the terminally differentiated phenotype CD28−CD57+ had impaired responses to IFN-α [19].

Prospective study revealed that CD4+CD45RO+ lymphocyte telomere lengths predicted the response to antiviral therapy in chronic HCV infection, independent of age, sex, HCV genotype, BMI and hepatic fibrosis, all factors that have been identified previously as predicting the response to antiviral therapy [21,29]. Our findings suggest that individuals with mild disease, but short telomeres should be considered for antiviral therapy at the earliest opportunity rather than adopting a conservative ‘watch and wait’ approach.

## Funding

M.H.: Wellcome Trust Clinical Research Training Fellow. W.G.: British Transplantation Society Research Fellow. A.D.: MRC Ph.D. student. The Frank Litchfield Charitable Trust and the Addenbrooke’s Hepatology Endowment Fund provided support for consumables. Roche pharmaceuticals provided an unconditional educational grant. The funders had no role in study design, data collection and analysis, decision to publish, or preparation of the manuscript.

## Conflicts of interest

The authors who have taken part in this study declared that they do not have anything to disclose regarding funding or conflict of interest with respect to this manuscript.

## Figures and Tables

**Fig. 1:**
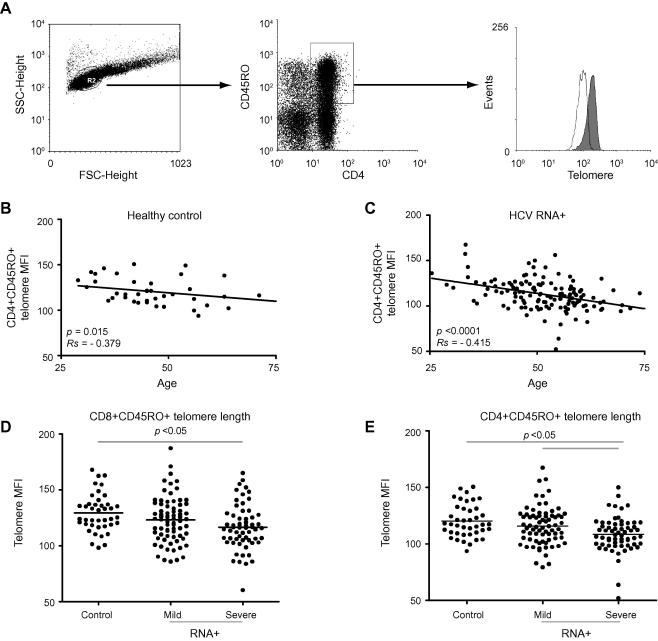
**Telomere length of CD8+ and CD4+ T-cells from healthy controls and HCV-infected subjects**. (A) Example of flow-FISH plots and gating strategy for analysis of telomere length. Left: Live lymphocyte gate by light-scatter characteristics. Centre: Identification of CD4+CD45RO+ cells. Right: Histograms of CD4+CD45RO+ lymphocyte telomere length from a subject with mild (filled histogram) and a subject with severe (unfilled histogram) fibrosis related to HCV infection. (B) The correlation between age and telomere length in circulating CD8+CD45RO+ lymphocytes from 41 healthy controls and (C) 135 HCV-infected subjects. (D) Telomere length of CD8+CD45RO+ lymphocytes in 41 healthy controls, 73 viraemic patients with mild fibrosis, and 61 viraemic patients with severe fibrosis. Horizontal bar represents the median. (E) Telomere length of CD4+CD45RO+ lymphocytes in 41 healthy controls, 73 viraemic patients with mild fibrosis, and 62 viraemic patients with severe fibrosis. Analysis by Kruskal–Wallis with Dunn’s multiple comparison test; bars show *p *<0.05.

**Fig. 3:**
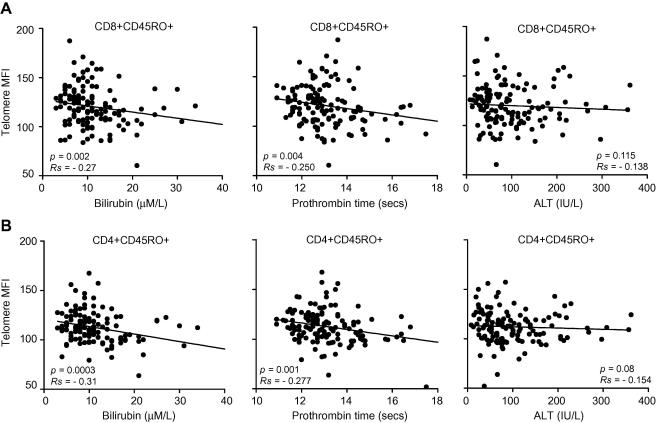
**Correlation of telomere length of (A) CD8+CD45RO+ or (B) CD4+CD45RO+ lymphocytes from 133 viraemic HCV patients with measures of the severity of liver disease: left panels, serum bilirubin (μM/L), middle panels, prothrombin time (seconds), and right panels, serum alanine transaminase (ALT) (IU/L)**.

**Fig. 5:**
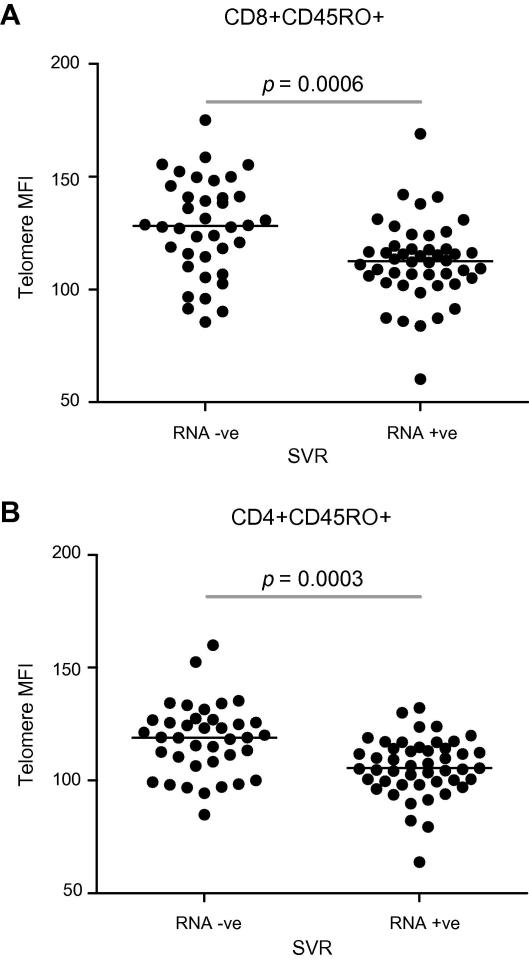
**Baseline T-lymphocyte telomere length in (A) CD8+CD45RO+ and (B) CD4+CD45RO+ subsets in relation to SVR (*n* = 85) following peg-IFNα and RBV**. Analysis by Mann–Whitney *U* test.

**Table 1:** **Demographic characteristics of subjects in the three study groups**.

	Healthy Controls	HCV-RNA positive mild	HCV-RNA positive severe	*p* value^†^
n	41	73	62	
Age (years, mean ± SD)	47.0 ± 11.1	49.4 ± 11.2	53.2 ± 7.2	**0.002**
Sex (% male)	51.2	71.2	77.4	0.12^1^
BMI (mean ± SD)	26.8 ± 3.5	25.6 ± 4.3	27.2 ± 5.6	0.19
Source of HCV:				
IDU%		58.9	67.7	0.40^1^
Blood products%		15.1	8.1	
Unknown%		26.0	24.2	
% IgG anti-HBc positive	12.2	26.0	32.3	**0.004^1^**
% CMV antibody positive	26.8	49.3	58.1	**0.007^1^**
Biochemical indices				
Bilirubin (*μ*mol/L, mean ± SD)	-	9.9 ± 5.1	14.3 ± 10.1	**0.009^2^**
ALT (IU/L, mean ± SD)	-	95.5 ± 88.7	109.1 ± 66.4	**0.02^2^**
PT (seconds, mean ± SD)	-	12.5 ± 0.8	13.8 ± 1.7	**<0.0001^2^**
Ishak score				
Interface hepatitis (0 - 4)	-	1.1 ± 0.8	1.9 ± 0.6	**<0.0001^2^**
Confluent necrosis (0 - 6)	-	0.0 ± 0.2	0.0 ± 0.2	**0.93^2^**
Lobular hepatitis (0 - 4)	-	1.8 ± 0.6	2.2 ± 0.6	**0.0007^2^**
Portal inflammation (0 - 4)	-	1.8 ± 0.7	2.3 ± 0.5	**0.0001^2^**
Fibrosis (0 - 6)	-	1.8 ± 0.9	4.8 ± 0.7	**<0.0001^2^**
Steatosis (0 – 3)	-	0.5 ± 0.7	0.9 ± 0.9	**0.002^2^**

Bold indicates significant results. ^†^Kruskal–Wallis unless otherwise stated: ^1^Chi-squared. ^2^Mann–Whitney *U* Test.

**Table 2:** **Predictors of severe fibrosis (Ishak fibrosis 4–6) by backward stepwise multinomial regression**. Input variables were gender, previous CMV or HBV, age, BMI, CD4+CD45RO+ telomere length, and CD8+CD45RO+ telomere length. Variables associated with severe fibrosis (*p *<0.1) in simple linear regression were included in a multiple regression analysis.

Variable	Simple multinomial regression					Backward stepwise multinomial regression				
	β	SE	df	OR (95% CI)	*p*	β	SE	df	OR (95% CI)	*p*
Male gender	0.89	0.44	1	2.43 (1.03, 5.74)	**0.043**	1.04	0 .50	1	2.84 (1.07, 7.53)	**0.04**
CMV+	1.33	0.44	1	3.78 (1.61, 8.83)	**0.002**	1.43	0 .48	1	4.18 (1.63, 10.73)	**0.003**
Age	0.07	0.02	1	1.08 (1.03, 1.12)	**0.001**					
BMI	0.02	0.04	1	1.02 (0.94, 1.10)	0.71					
Anti-HBc+	1.65	0.54	1	5.20 (1.80, 15.1)	**0.002**	1.58	0 .58	1	4.74 (1.53, 14.72)	**0.007**
CD4+CD45RO+ telomere length	0.05	0.01	1	1.05 (1.02, 1.08)	**0.001**	0.04	0 .01	1	1.05 (1.02, 1.78)	**0.003**
CD8+CD45RO+ telomere length	0.03	0.01	1	1.03 (1.01, 1.06)	**0.002**					

Bold indicates significant results.

**Table 3:** **Unadjusted and adjusted Hazard Ratios (and 95% CI) for decompensation, development of HCC and a composite endpoint of death, decompensation or development of HCC by CD8+CD45RO+ and CD4+CD45RO+ T-lymphocyte telomere length and age in whole viraemic cohort (*n* = 124) or subjects with severe fibrosis (*n* = 55)**.

Bold indicates significant results.

**Table 4:** Demographic characteristics of interferon-treated cohort.

	Whole cohort	Failed to achieve SVR	Achieved SVR
n	85	47	38
Age (years, mean ± SD)	52.3 ± 8.8	55.0 ± 8.9	49.7 ± 7.6
Sex (% male)	75.3	72.3	78.9
BMI	26.6 ± 5.3	27.5 ± 5.7	25.5 ± 4.5
Bilirubin (μmol/L, mean ± SD)	12.3 ± 6.6	12.2 ± 6.1	11.5 ± 6.8
ALT (IU/L, mean ± SD)	113.2 ± 81.9	112.7 ± 83.2	117.1 ± 85.7
PT (seconds, mean ± SD)	13.2 ± 1.4	13.2 ± 1.1	13.2 ± 1.7
Genotype 1	45.9%	53.2%	36.8%
2	11.8%	10.6%	13.2%
3	41.2%	36.2%	47.4%
4	1.1%	0%	2.6%
Viral Load (mean ± SD)	1.35 × 10^6^ ± 6.99 × 10^6^	2.31 × 10 ^6^ ± 9.34 × 10^6^	1.67 × 10^5^ ± 2.54 × 10^5^
% CMV antibody positive	51.0	51.1	50.0
Ishak score			
Interface hepatitis (0 - 4)	1.8 ± 0.6	1.7 ± 0.5	1.8 ± 0.7
Confluent necrosis (0 - 6)	0.1 ± 0.2	0.1 ± 0.3	0.0 ± 0.2
Lobular hepatitis (0 - 4)	2.2 ± 0.6	1.0 ± 0.5	2.3 ± 0.6
Portal inflammation (0 - 4)	2.2 ± 0.6	2.2 ± 0.6	2.2 ± 0.6
Fibrosis (0 - 6)	3.6 ± 1.5	3.9 ± 1.4	3.4 ± 1.5
Steatosis (0 - 3)	0.8 ± 0.8	1.0 ± 0.8	0.6 ± 0.8

**Table 5:** **Predictors of SVR by multiple logistic regression analysis**. Input variables were age, gender, fibrosis group (mild (Ishak 0–3), severe (4–6)), viral genotype (2 and 3 versus 1 and 4), viral load, BMI, CD8+CD45RO+ and CD4+CD45RO+ lymphocyte telomere length. Non-significant variables were removed by backward stepwise regression.

	Logistic regression				Backward LR stepwise regression			
Variable	Comparison	Wald	OR (95% CI)	*p*	Comparison	Wald	OR (95% CI)	*p*
Age	-	0.72	0.97 (0.89, 1.05)	0.40				
Sex	Male vs. female	0.53	1.74 (0.39, 7.66)	0.47				
Fibrosis	Severe vs. Mild	3.03	0.18 (0.03, 1.24)	0.08				
Genotype	2/3 vs. 1/4	1.90	2.17 (0.71, 6.89)	0.17				
Viral load	-	5.74	1.00 (1.00, 1.00)	**0.02**	-	5.58	1.00 (1.00, 1.00)	**0.02**
BMI	-	0.007	1.00 (0.88, 1.12)	0.93				
CD8+45RO+ telomere length	-	0.09	1.01 (0.96, 1.06)	0.76				
CD4+45RO+ telomere length	-	2.59	1.06 (0.99, 1.14)	0.11	-	11.28	1.08 (1.03, 1.13)	**0.001**

Bold indicates significant results.
